# Achieving quality primary care data: a description of the Canadian Primary Care Sentinel Surveillance Network data capture, extraction, and processing in Alberta

**DOI:** 10.23889/ijpds.v4i2.1132

**Published:** 2019-07-29

**Authors:** S Garies, M Cummings, B Forst, K McBrien, B Soos, M Taylor, N Drummond, D Manca, K Duerksen, H Quan, T Williamson

**Affiliations:** 1 Department of Family Medicine, University of Calgary, G012 Health Sciences Centre, 3330 Hospital Drive NW, Calgary Alberta, Canada, T2N 4N1; 2 Department of Community Health Sciences, University of Calgary, 3280 Hospital Drive NW, Calgary, Alberta, Canada, T2N 4Z6; 3 Department of Family Medicine, University of Alberta, 6-10 University Terrace, Edmonton, Alberta, Canada, T6G 2T4

## Abstract

**Introduction:**

Electronic medical record (EMR) databases have become increasingly popular for secondary purposes, such as health research. The Canadian Primary Care Sentinel Surveillance Network (CPCSSN) is the first and only pan-Canadian primary care EMR data repository, with de-identified health information for almost two million Canadians. Comprehensive and freely available documentation describing the data ‘lifecycle’ is important for assessing potential data quality issues and appropriate interpretation of research findings. Here, we describe the flow and transformation of CPCSSN data in the province of Alberta.

**Approach:**

In Alberta, the data originate from 54 publicly-funded primary care settings, including one community pediatric clinic, with 318 providers contributing de-identified EMR data for 410,951 patients (as of December 2018). Data extraction methods have been developed for five different EMR systems, and include both backend and automated frontend extractions. The raw EMR data are transformed according to specific rules, including trimming implausible values, converting values and free text to standard terminologies or classification systems, and structuring the data into a common CPCSSN format. Following local data extraction and processing, the data are transferred to a central repository and made available for research and disease surveillance.

**Conclusion:**

This paper aims to provide important contextual information to future CPCSSN data users.

## Introduction

The proportion of family physicians using electronic medical record (EMR) systems has been steadily increasing in Canada, with an estimated 85% reporting some degree of EMR use in practice in 2017 [1]. Coupled with advancements in computing power, data storage capacity, and analytic methods for large datasets, primary care EMRs are becoming a popular source of data for health research and other secondary uses [2, 3]. The Canadian Primary Care Sentinel Surveillance Network (CPCSSN) is a pan-Canadian primary care EMR database made available for health research, disease surveillance, and clinical quality improvement [3, 4]. The CPCSSN data have been used to develop and validate case definitions for many conditions relevant to primary care [5-7], report on the epidemiology of chronic conditions across Canada [8-15], contribute to EMR data methodology and quality [16-19], and conduct various health outcomes research [20-24]. However, in order to ensure the CPCSSN data can be used for these purposes, sophisticated cleaning and processing techniques are required to transform the raw EMR data extracted from primary care settings into useable information. This includes coding free text, trimming implausible values, and standardizing the data into a common format. The complexity of these tasks becomes multiplied due to variation in data quality and quantity between providers, clinics, provinces/territories, and EMR systems.

Comprehensive documentation describing the data flow is a critical part of ensuring high data quality; more specifically, understanding where the data originate, the process for extraction and transformation, and how data samples are created for analysis. Without this information, it would be challenging to determine whether the data are suitable for the intended use and any research findings would risk misinterpretation. The Kahn et al. framework for reporting data quality in distributed networks provides a template for thoroughly describing the entire lifecycle of data, while aligning with several STROBE (Strengthening the Reporting of Observational Studies in Epidemiology) recommendations [25]. Although an overview of the CPCSSN organization and database has been previously published [4], a detailed description of the data capture and processing outlined in the Kahn framework have not, to date, been available for the CPCSSN database. The purpose of this paper is to provide a more technical report of the generation, extraction, and processing of primary care EMR data in Alberta. We anticipate that this work will support prospective users in their understanding of the CPCSSN data context and quality.

## Approach

### Original Data Source

Canada has a universal, publicly funded healthcare system with each province or territory managing the delivery of healthcare according to their own jurisdiction’s needs. Because of this, variation exists between each province and territory, in terms of health information legislation, compensation models for physicians, medications available for prescribing, and healthcare organization. Primary care is considered the first point of contact for patients in the health system and consists of medical care, preventive services, chronic disease management, and referral to specialist physicians. In Alberta, primary care providers often work in multi-disciplinary team practices that include family physicians and other allied health professionals such as nurses, nurse practitioners, dietitians, mental health specialists, and many others. Patients are free to visit any primary care practice of their choosing at any time, although attempts have been made to formally attach people to a singular primary care provider to promote better continuity of care. During a primary care consultation, the patient’s relevant information is entered into the EMR system, usually manually, though exactly what is recorded depends on provider preferences and EMR system input options. Generally, information such as demographics, diagnoses (or symptoms or suspected conditions), prescribed medications, physical measurements (such as blood pressure, height, weight), risk factors (smoking, alcohol use), allergies, vaccinations, and medical procedures are entered by the clinicians or by clinic staff (e.g. physician, nurse, receptionist, clerk). Results from laboratory service providers are electronically merged into the patient record.

In 2008, the Public Health Agency of Canada funded the initial development of CPCSSN, with the goal of creating a pan-Canadian repository of primary care EMR data that could be used for surveillance and research. At the present time, CPCSSN has grown to over 1200 primary care providers from eight provinces and territories who contribute de-identified data for close to two million patients [26]. CPCSSN is a collaboration of eleven regional primary care practice-based research networks (PBRN) across Canada. Primary care PBRNs are typically multidisciplinary collaborations between family physicians, allied health care providers, and university-affiliated primary care researchers with the purpose of addressing questions deriving from clinical practice and facilitating improvement in primary care settings. Each PBRN recruits ‘sentinel’ primary care providers (family physicians, nurse practitioners) and community pediatricians to the project and is also responsible for local extraction, cleaning, and processing of the EMR data prior to transferring the regional data to the central repository located at Queen’s University in Kingston, Ontario [4]. Additional details about the organization and data processes of the national CPCSSN database have been described elsewhere [4, 26]. Across the country, CPCSSN actively extracts data from eleven different EMR systems, which vary by region; however, this paper is focused on EMR data obtained by the two regional PBRNs in the province of Alberta – the Northern and Southern Primary Care Research Networks (NAPCReN and SAPCReN, respectively). Between both Alberta networks, 318 providers from 53 primary care practices and one community pediatric clinic contribute de-identified data from 410,951 patients in the province, as of December 31, 2018 (supplementary file). This represents just over 5% of the total population of family physicians in Alberta [27].

### Data Stewardship

NAPCReN and SAPCReN are embedded within the Departments of Family Medicine at the University of Alberta (NAPCReN) and the University of Calgary (SAPCReN), and are funded through various project grants and departmental contributions. As outlined in Alberta’s health legislation, health care service providers, such as nurses and physicians, may be designated as custodians of the patient data. Both NAPCReN and SAPCReN have research ethics board approval for the CPCSSN project at their respective institutions. Through research agreements with each data custodial ‘sentinel’ provider, NAPCReN and SAPCReN are the data stewards who are permitted to extract and process de-identified EMR data before transferring to the central CPCSSN repository. Sentinels contribute to CPCSSN voluntarily, without financial compensation, and for their participation receive their panel data in the form of routine feedback reports and online tool for their participation, as well as membership in their respective PBRNs. Patients are informed of the CPCSSN project through information posters and brochures in each clinic, and are able to opt-out of the database at any time by contacting a PBRN staff member or informing their provider. If this does occur, the CPCSSN data manager will obtain a unique identifier associated with the patient’s record (EMR ID) from the clinic and add an exclusion for this ID to the extraction code.

### Data Extraction

The supplementary file provides details about the data extraction and processing. Alberta has one of the highest rates of EMR use in family practice of Canadian provinces and territories at >86% [1]. NAPCReN and SAPCReN have extraction processes for five EMR systems in Alberta: Wolf, Med Access, Practice Solutions Suite, Accuro, and Healthquest. Data extraction occurs every six months. The extraction process depends on the EMR system and whether they use a hosted/Application Service Provider (ASP) or a local/non-ASP system. Two systems require backend extractions through the vendor (Wolf, Practice Solutions Suite), two allow backend extraction by a CPCSSN data manager (Accuro, Healthquest), and one is an automated frontend extraction (Med Access). A full list of data elements extracted is available in the CPCSSN Data Dictionary [28].

An EMR-to-CPCSSN patient ID mapping file is also created at the time of extraction. This file contains the original EMR patient ID that is assigned to each patient within a clinic EMR system, the CPCSSN network ID and CPCSSN site ID for the clinic from which the data are extracted, and the unique CPCSSN patient ID that is randomly assigned to each patient. The file is stored separately from the de-identified health data and is used to ensure that, during data extraction, CPCSSN patient IDs are assigned uniquely and consistently to each EMR patient ID, as well as to facilitate re-identification for practice quality improvement or data linkage.

### Data Processing & Data Provenance

CPCSSN has developed a number of processing steps that transform raw EMR data into a format that is compatible with the CPCSSN database structure. Many elements in the CPCSSN database contain both an ‘original’ field and a CPCSSN ‘calculated’ or ‘coded’ field. At the regional level, specific processing work is undertaken following data extraction, including general data cleaning for specific fields (e.g. standardizing dates, deleting empty or duplicate fields), trimming values to fit allowable ranges, mapping original data to standard classification or coding systems, and some additional transformations to create new variables (see supplementary file). Each CPCSSN-affiliated PBRN may have different processes for the transformation of their local data; due to shared data manager resources between NAPCReN and SAPCReN, the processes described here are the same for both Alberta networks unless otherwise specified in the supplementary file.

### Mapping from original values to standardized values

Standardization of numerical CPCSSN data is relatively straightforward. For physical examination or laboratory data, all values are converted to one common set of units. Bounds are also placed on the data, and values that lie beyond these ranges are not coded into the database. Age and date ranges are also restricted. Any age or year entry (e.g. patient birth year) must indicate that the patient is less than or equal to 120 years old at the time of data extraction. Dates are constrained by type: those relating to EMR record creation date must fall within the range of 1990-01-01 to the extraction date, while condition onset or procedure performed dates must be between the extraction date minus 120 years and the extraction date.

The original EMR data are mapped to standard classification or coding systems, such as Anatomical Therapeutic Chemical (ATC) codes for medication, International Classification of Disease version nine (ICD-9) codes for diagnostic text, and Logical Observation Identifiers Names and Codes (LOINC) for laboratory values. Unstructured risk factor information (such as smoking and alcohol use) is categorized into risk type (and additional categories, if the data are present) using a pattern matching approach. Some additional transformations are performed to create new variables, including BMI calculated from weight and height (if no BMI exists), and disease cases indexed according to CPCSSN’s validated definitions [5]. Material and social deprivation quintiles based on mapping postal codes to the Pampalon Index [29] will be integrated into the database in the near future.

### Data processing validation

To validate the transformation processes, both manual and automated routines are applied to the CPCSSN-produced data. Consistency and completeness of extracted data are analyzed through manual file size and number checks. Automated software-based analysis can also be applied, including calculation of per provider statistics (number per clinic, number of patients, number of records) and comparison to values from the previous extraction. Transformation completeness is primarily assessed through review of the verbose log files. These files contain initial and final row counts for each table in the database, and the numbers of rows affected by each process applied to the data. Data generated following the coding/cleaning processes are also assessed by manual viewing of log files; however, the underlying software first undergoes two other tests. To determine how updates to specific coding and/or cleaning algorithms will affect data processing, the software is run against algorithm-specific CPCSSN reference data standards. For example, if changes are made to the exam cleaning algorithm, the software is run on a copy of the exam reference data set. The resulting cleaned data are then compared to the original exam reference set and any changes are assessed. Additionally, an end-to-end test is applied, where all steps of the coding/cleaning software are run on the gold standard data set that was used to define the original eight CPCSSN disease case definitions [5]. The sensitivity, specificity, positive predictive value (PPV), and negative predictive value (NPV) for these eight diseases are then calculated and compared to values produced by the previous version of the software.

Once a database has been coded, cleaned, and all case detection algorithms run, personal identifiers that are imbedded in free-text are redacted. This is accomplished through matching to eight classes of regular expression patterns, including patient first and last names, provincial health numbers, and phone numbers. For a list of redacted terms, see supplementary file.

### Audit trail

Verbose logging is produced at each of the three processing stages (extraction, transformation, and coding/cleaning) which serves as an audit trail. One extraction and one transformation log are produced per clinic. Following the transformation stage, databases for all clinics within a network are generally merged, and so there is typically only a single coding/cleaning log for each network. Each line of the log files begins with a timestamp, containing the current date and time, and a message that may contain processing software version information, source or destination file path or numbers of data elements affected by a process. Warning and error messages, including information such as missing source files, processes that failed, or data elements that are beyond permitted ranges, are also recorded.

Each PBRN typically completes data processing on their own secure server that is housed at the Centre for Advanced Computing (CAC) at Queen’s University. Once the CPCSSN data are processed and standardized, the data are transferred within the secure network at the CAC to the central CPCSSN repository through simple file transfer. Data from all networks are then combined within the central repository to form the national CPCSSN database.

## Discussion

### Summary of What This Paper Adds

**Figure 1: Potential sources of bias and data quality issues in Canadian primary care EMR data. (adapted from Verheij et al. [30]):**
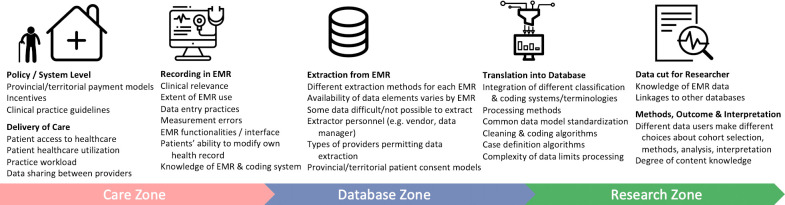


We have described the process used by NAPCReN and SAPCReN for data extraction, transformation, cleaning/coding, and merging, with the aim of providing important context for current and future users of the CPCSSN data. In the full lifecycle of EMR data, there are many potential opportunities for data quality issues and biases to occur [30]. Particularly in distributed data networks, variability may occur due to differences in provincial/territorial policies, payment models, delivery of care, availability of EMR systems, and healthcare utilization, among others (Figure 1). Additionally, the local extraction and processing for CPCSSN can vary by regional PBRN, which underscores the importance of having detailed documentation available for data users. This work offers a set of standards that may be used by other PBRNs to describe their own EMR data practices and/or to adopt the procedures described here.

**Figure 2: CPCSSN Data Pipeline:**
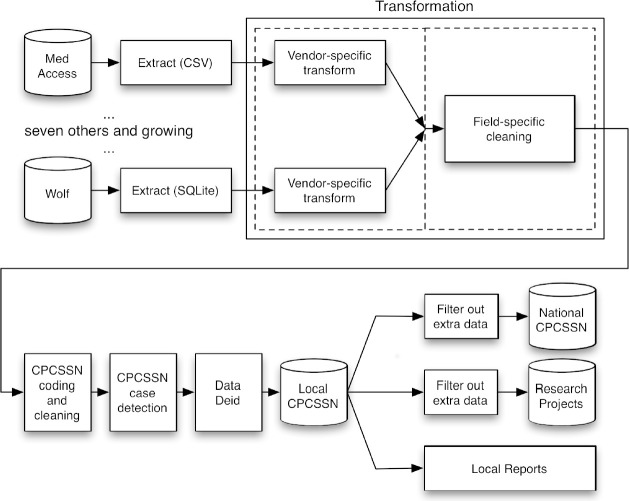


### Lessons Learned

The CPCSSN data extraction and transformation processes have been achieved through more than a decade of dedicated work by a national team of data managers. Significant advantages of the CPCSSN data (compared to the source EMR data) include implementation of many automated processes for cleaning raw data and mapping of values or text to standard classification systems (such as the ATC Classification System, LOINC, and ICD-9). Additionally, the development and implementation of 15 (and counting) validated case definitions allow clinicians and researchers to more accurately identify patients with certain conditions. Informal searching through poorly coded or unstructured raw data limits the ability of clinicians to undertake quality improvement projects or introduces biases when conducting research or surveillance. The CPCSSN team places significant importance on having valid case definitions for the CPCSSN database, as demonstrated by an internal ‘Case Definition Working Group’ and its efforts to continually evaluate and improve existing definitions, while working towards the development of new ones.

Collaboration between regional networks and the central CPCSSN data management office at Queen’s University has been essential for the coordination of CPCSSN data-focused tasks, though this is not always easy to achieve. In particular, sound software development principles are vital in developing and coordinating robust extraction, transformation, and coding/cleaning tools between eleven pan-Canadian networks. Use of a version control system, such as GIT or Mercurial, and tagging of software versions is essential, so that it is clear precisely which version of a given program, plus any associated configuration files, were used to generate a particular data set. Increasing the level of automation to reduce the amount of direct human intervention in the processing pipeline has greatly increased the level of data consistency and reproducibility not only between clinics, but also between extraction cycles. EMR data are surprisingly messy, so software developed to process it quickly becomes complex. Since even carefully written code will contain bugs, by developing test data sets and testing software, coding errors are more easily caught and corrected. Finally, while machine learning methods can be extremely powerful tools for data cleaning and coding, they are not always the best solution. In many cases, simple pattern matches can be implemented more quickly and with comparable or higher accuracy, and can lead to more maintainable software.

A sample of the national CPCSSN database is available to approved university-affiliated researchers after submission of a letter of intent for review by the CPCSSN research committee, research ethics approval at their host university, and payment of data access fees; more details are provided on the CPCSSN website (www.cpcssn.ca). Access to regional CPCSSN data may be permitted by the director of the PBRN upon discussion, research ethics approval (for research studies) and adherence to any network-specific requirements.

### Ongoing Development / Future Work

The extraction and transformation processes described here will undoubtedly evolve as CPCSSN continues to recruit primary care providers and expand its data holdings. Future plans include improvements to the risk factor classifier algorithms (e.g. more accurately assigning patients to the correct smoking status), additional coding for diagnoses entered as free text, and exploration of extraction methods for new EMR systems. Machine learning-based improvements to lab, medication and referral coding are also underway.

Linkage of the CPCSSN EMR data to administrative data sources (e.g. hospitalizations, emergency department visits, pharmacy dispensed medications) is a major step towards a more complete understanding of a patient’s journey through the health care system. Although the CPCSSN data are de-identified, a mapping file generated from each clinic EMR system (containing EMR ID, patient Personal Health Number (PHN), date of birth and sex) can facilitate linkage based on PHN with administrative data held in the provincial health authority’s data warehouse. A second mapping file from CPCSSN (which includes patient EMR ID and CPCSSN patient ID) can be used to map the de-identified CPCSSN data to the administrative data using EMR ID. Each regional PBRN may have a different process for data linkage (if at all), largely due to differences in the privacy laws and health information acts within each province/territory. For those PBRNs that conduct data linkage, the CPCSSN EMR data are linked and held within each province/territory and are not part of the national CPCSSN database.

### Limitations

This paper describes CPCSSN data from two regional PBRNs in Alberta that collect de-identified EMR data from a sample of primary care providers, which may not reflect the characteristics or practice of all providers in Alberta or of the 37,000+ family physicians [27] in other provinces/territories. Although citizens who are registered with the health care system in each province/territory are assigned a unique health identifier (termed Personal Health Number (PHN) in Alberta), identifiable information (including PHN) are not collected as part of the CPCSSN database. This means that CPCSSN currently has no way to identify patients who receive care at multiple clinics and thus, may be assigned multiple CPCSSN IDs. While progress on rostering patients within Alberta to a specific primary care provider may help to solve this problem, the extent of the issue in the CPCSSN data for Alberta is not known.

Primary care EMR data in Canada are still a relatively new source of health information and there are few existing standards for EMR data entry, extraction, and processing. Thus, some of the processing steps were developed in response to a need at a particular time, rather than based on an established reference standard. Any process that is manually-driven may be prone to error and lessens the ability to replicate work. In addition, this paper is limited to the description of extraction and processes in Alberta, which may vary from the other CPCSSN-contributing PBRNs across Canada. Given the distributed nature of the national CPCSSN project and the scarcity of process documentation available to data users, it is hoped that this work serves as a template for PBRNs to record their data capture and transformation processes. Lastly, both recruitment of CPCSSN primary care providers in Alberta and updates to the technical cleaning and coding work is ongoing, meaning that the information provided here may change over time as providers enter or exit the sample and data processing improvements are implemented.

EMR data quality is likely to vary based on data capture; this is related to data entry practices in each clinic by different providers and how well the EMR interface facilitates the recording of information. At present, we do not capture any details about variations in clinical data entry and design of EMR systems. Furthermore, NAPCReN and SAPCReN do not extract healthcare provider notes (e.g. ‘Subjective, Objective, Assessment, and Plan [SOAP] notes) or PDF documents, such as referral letters or diagnostic imaging.

## Conclusion

Primary care EMR data are a valuable source for surveillance and health research, in addition to facilitating more rapid clinical quality improvement and evaluation. There are many complex steps needed to extract and transform raw EMR data into useable information, which have been described here. The comprehensive documentation of the CPCSSN processes in Alberta will enable better understanding and use of these nuanced data, as well as provide a systematic methodology that can be used by other groups working in this domain.

## Supplementary File



## Abbreviations

ATC	Anatomical Therapeutic Chemical [classification system]
BMI	Body Mass Index
CPCSSN	Canadian Primary Care Sentinel Surveillance Network
DBP	Diastolic Blood Pressure
DIN	Drug Identification Number
EMR	Electronic Medical Record
ERD	Entity Relationship Diagram
FSA	Forward Sortation Area
ICD-9	International Classification of Disease version 9
LOINC	Logical Observation Identifiers Names and Codes
NAPCReN	Northern Alberta Primary Care Research Network
PBRN	Practice-based Research Network
PDF	Portable Document Format
PEFR	Peak Expiratory Flow Rate
SAPCReN	Southern Alberta Primary Care Research Network
SBP	Systolic Blood Pressure
SFTP	Secure File Transfer Protocol
SNOMED	Systematized Nomenclature of Medicine
SQL	Structured Query Language

## References

[1] Canadian Medical Association. CMA Physician Workforce Survey, 2017. National Results by FP/GP or Other Specialist, Gender, Age, and Province/Territory. CMA Physician Workforce Survey 2017. https://surveys.cma.ca/en/permalink/survey31. Published 2017. Accessed July 15, 2019.

[2] Kruse CS, Goswamy R, Raval Y, Marawi S. Challenges and opportunities of big data in health care: a systematic review. JMIR Med Informatics. 2016;4(4):e38. 10.2196/medinform.5359 PMC5138448 27872036

[3] Birtwhistle R, Williamson T. Primary care electronic medical records: A new data source for research in Canada. CMAJ. 2015;187(4):239-240. 10.1503/cmaj.140473 25421989 PMC4347766

[4] Garies S, Birtwhistle R, Drummond N, Queenan J, Williamson T. Data Resource Profile: National electronic medical record data from the Canadian Primary Care Sentinel Surveillance Network (CPCSSN). Int J Epidemiol. 2017;46(4):1091-1092f. 10.1093/ije/dyw248 28338877

[5] Williamson T, Green ME, Birtwhistle R, et al. Validating the 8 CPCSSN case definitions for chronic disease surveillance in a primary care database of electronic health records. Ann Fam Med. 2014;12(4):367-372. 10.1370/afm.1644 25024246 PMC4096475

[6] Cave AJ, Davey C, Ahmadi E, et al. Development of a validated algorithm for the diagnosis of paediatric asthma in electronic medical records. NPJ Prim Care Respir Med. 2016;26:16085. 10.1038/npjpcrm.2016.85 27882997 PMC5122312

[7] Lethebe BC, Williamson T, Garies S, et al. Developing a case definition for type 1 diabetes mellitus in a primary care electronic medical record database: an exploratory study. CMAJ Open. 2019;7(2):E246-E251. 10.9778/cmajo.20180142 PMC6504632 31061005

[8] Godwin M, Williamson T, Khan S, et al. Prevalence and management of hypertension in primary care practices with electronic medical records: a report from the Canadian Primary Care Sentinel Surveillance Network. CMAJ Open. 2015;3(1):E76-E82. 10.9778/cmajo.20140038 PMC4382047 25844373

[9] Greiver M, Williamson T, Barber D, et al. Prevalence and epidemiology of diabetes in Canadian Primary Care Practices: A report from the Canadian Primary Care Sentinel Surveillance Network. Can J Diabetes. 2014;38(3):179-185. 24835515 10.1016/j.jcjd.2014.02.030

[10] Williamson T, Green ME, Jordan KP, et al. Prevalence and management of osteoarthritis in primary care: an epidemiologic cohort study from the Canadian Primary Care Sentinel Surveillance Network. CMAJ Open. 2015;3(3):E270-E275. 10.9778/cmajo.20150018 PMC4593417 26442224

[11] Williamson T, Natajaran N, O’Donnell DE, et al. Chronic obstructive pulmonary disease in primary care: an epidemiologic cohort study from the Canadian Primary Care Sentinel Surveillance Network. CMAJ Open. 2015;3(1):E15-E22. 10.9778/cmajo.20140040 PMC4382041 25844366

[12] Williamson T, Khan S, Manca D, et al. The diagnosis of depression and its treatment in Canadian primary care practices: an epidemiological study. CMAJ Open. 2014;2(4):E337-E342. 10.9778/cmajo.20140052 PMC4251512 25485260

[13] Khan S, Garies S, Drummond N, Molnar F, Birtwhistle R, Williamson T. Prevalence and management of dementia in primary care practices with electronic medical records: a report from the Canadian Primary Care Sentinel Surveillance Network. CMAJ Open. 2016;4(2):E177-E184. 10.9778/cmajo.20150050 PMC4933596 27398361

[14] Queenan JA, Farahani P, Ehsani-Moghadam B, Birtwhistle R V. The prevalence and risk for herpes zoster infection in adult patients with diabetes mellitus in the Canadian Primary Care Sentinel Surveillance Network. Can J Diabetes. 2018;42(5):465-469. 29395844 10.1016/j.jcjd.2017.10.060

[15] Rigobon A V, Birtwhistle R, Khan S, et al. Adult obesity prevalence in primary care users: An exploration using Canadian Primary Care Sentinel Surveillance Network (CPCSSN) data. Can J Public Health. 2015;106(5):e283-e289. 10.17269/CJPH.106.4508 26451989 PMC6972402

[16] Queenan JA, Williamson T, Khan S, et al. Representativeness of patients and providers in the Canadian Primary Care Sentinel Surveillance Network: a cross-sectional study. CMAJ Open. 2016;4(1):e28-e32. 10.9778/cmajo.20140128 PMC4866925 27331051

[17] Greiver M, Williamson T, Bennett TL, et al. Developing a method to estimate practice denominators for a national Canadian electronic medical record database. Fam Pract. 2013;30(3):347-354. 10.1093/fampra/cms083 23307818

[18] Greiver M, Barnsley J, Aliarzadeh B, et al. Using a data entry clerk to improve data quality in primary care electronic medical records: a pilot study. Inform Prim Care. 2011;19(4):241-250. 10.14236/jhi.v19i4.819 22828579

[19] Singer A, Yakubovich S, Kroeker AL, Dufault B, Duarte R, Katz A. Data quality of electronic medical records in Manitoba: Do problem lists accurately reflect chronic disease billing diagnoses? J Am Med Inform Assoc. 2016;23(6):1107-1112. 10.1093/jamia/ocw013 PMC11960764 27107454

[20] Greiver M, Kalia S, Voruganti T, et al. Trends in end digit preference for blood pressure and associations with cardiovascular outcomes in Canadian and UK primary care: A retrospective observational study. BMJ Open. 2019;9(1):1-11. 10.1136/bmjopen-2018-024970 PMC6347875 30679298

[21] Greiver M, Dahrouge S, O’Brien P, et al. Improving care for elderly patients living with polypharmacy: Protocol for a pragmatic cluster randomized trial in community-based primary care practices in Canada. Implement Sci. 2019;14(1). 10.1186/s13012-019-0904-4 PMC6551894 31171011

[22] Reyes RR, Parker G, Garies S, et al. Team-based comanagement of diabetes in rural primary care. Can Fam Physician. 2018;64(8). PMC6189896 30108089

[23] Garies S, Irving A, Williamson T, Drummond N. Using EMR data to evaluate a physician-developed lifestyle plan for obese patients in primary care. Can Fam Physician. 2015;61(5):e225-e231. 26167562 PMC4430071

[24] Brown F, Singer A, Katz A, Konrad G. Statin-prescribing trends for primary and secondary prevention of cardiovascular disease. Can Fam Physician. 2017;63(11):e495-e503. 29138175 PMC5685466

[25] Kahn MG, Brown JS, Chun AT, et al. Transparent reporting of data quality in distributed data networks. EGEMS (Washington, DC). 2015;3(1):Article 7. 10.13063/2327-9214.1052 PMC4434997 25992385

[26] CPCSSN. Canadian Primary Care Sentinel Surveillance Network (CPCSSN). www.cpcssn.ca. Published 2016. Accessed February 14, 2019.

[27] Canadian Medical Association. Family Medicine Profile. 2018. https://www.cma.ca/sites/default/files/family-e.pdf.

[28] Canadian Primary Care Sentinel Surveillance Network. CPCSSN Data Dictionary and ERD. http://cpcssn.ca/research-resources/cpcssn-data-dictionary-and-erd/. Published 2016. Accessed May 23, 2019.

[29] Gamache P, Hamel D, Pampalon R. The material and social deprivation index: a summary. https://www.inspq.qc.ca/sites/default/files/santescope/indice-defavorisation/en/GuideMethodologiqueEN.pdf. Published 2017. Accessed November 26, 2018.

[30] Verheij RA, Curcin V, Delaney BC, McGilchrist MM. Possible sources of bias in primary care electronic health record data use and reuse. J Med Internet Res. 2018;20(5):e185. 10.2196/jmir.9134 29844010 PMC5997930

[31] Murray M, Davies M, Boushon B. Panel size: how many patients can one doctor manage? Fam Pract Manag. 2007;14(4):44-51. http://www.ncbi.nlm.nih.gov/pubmed/17458336. 17458336

