# Supplementary material for: Achieving quality primary care data: a description of the Canadian Primary Care Sentinel Surveillance Network data capture, extraction, and processing in Alberta
Source: Int J Popul Data Sci. 2019 Jul 29;4(2):1132. doi: 10.23889/ijpds.v4i2.1132 (PMC8142949; doi:10.23889/ijpds.v4i2.1132)
Supplement: Supplementary file 1 [file ijpds-04-1132-s001.pdf]

Supplementary File.
Data Quality Assessment Documentation and Reporting Recommendations (as outlined by Khan [25])

<table>
<tr><th colspan="3">Data Capture</th></tr>
<tr><td colspan="3">Original Data Source</td></tr>
<tr><td>Data origin</td><td>NAPCReN:
EMR data from primary care clinical practices in northern Alberta using one of Accuro (n=1), Healthquest (n=1), Med Access (n=11), or Wolf (n=6) EMR system.

As of December 31, 2018, data was captured from:
19 primary care clinics
79 providers (family physicians)
104,622 patients</td><td>SAPCReN:
EMR data from primary care clinical practices in southern Alberta using one of Med Access (n=12), Practice Solutions (n=1), or Wolf (n=22) EMR system.

As of December 31, 2018, data was captured from:
34 primary care clinics + 1 community pediatric clinic
239 providers (family physicians, nurse practitioners, community pediatricians)
306,329 patients</td></tr>
<tr><td>Data capture method</td><td colspan="2">Direct entry by clinicians/clinical staff into EMR system; interface and available templates/data fields may vary by EMR system.

Automated upload of laboratory results into EMR from community lab provider.</td></tr>
<tr><td>Original collection purpose</td><td colspan="2">Patient clinical care and administrative tasks (i.e. billing) in community-based primary care clinics in Alberta.</td></tr>
<tr><td colspan="3">Data Steward Information</td></tr>
<tr><td>Data steward</td><td>NAPCReN:
Northern Alberta Primary Care Research Network (NAPCReN), one of 11 regional practice-based research networks hosting the CPCSSN project. Housed within the Department of Family Medicine, University of Alberta; Edmonton, Alberta, Canada.</td><td>SAPCReN:
Southern Alberta Primary Care Research Network (SAPCReN), one of 11 regional practice-based research networks hosting the CPCSSN project. Housed within the Department of Family Medicine, University of Calgary; Calgary, Alberta, Canada.</td></tr>
</table>

Database model/structure	The CPCSSN data are stored as a SQL database (either SQL Server or SQLite format) and currently contains 26 tables:<br>▪ Eight tables house clinic, health care provider, or administrative information (e.g. patient-provider assignment, non-identifiable clinic information, database version information)<br>▪ Fifteen tables hold both original EMR data and processed (cleaned and coded) data<br>▪ Two tables (DiseaseCase, DiseaseCaseIndicator) contain a list of patients who have been indexed with one or more of the conditions for which CPCSSN has a validated definition; these definitions use data derived from the 15 data tables<br>▪ The Deprivation table is empty, but will be populated in the future.<br><br>Each table is assigned an integer primary key labelled: <table_name>_ID. Most data tables also contain the columns:<br>▪ Site_ID (an integer assigned to each primary care practice) and Network_ID (an integer assigned to each PBRN) together form a composite primary key in the Site table, and a composite foreign key in most CPCSSN tables<br>▪ Patient_ID (randomly assigned integer for each patient) is the primary key in Patient table and a foreign key in most other tables<br>▪ Encounter_ID is the integer primary key for the Encounter table, but a foreign key in several other tables<br>▪ Provider_ID is the integer primary key for the Provider table and a foreign key for several tables<br>▪ All columns are strictly typed, enforced either by the database schema (SQL Server) or external software (SQLite)<br><br>The Entity Relationship Diagram (ERD), which outlines relationships between all CPCSSN tables, can be found on the CPCSSN website: http://cpcssn.ca/research-resources/cpcssn-data-dictionary-and-erd/<br><br>For SAPCReN and NAPCReN, extraction, transformation, coding, and cleaning algorithms are programmed in Python.
Data dictionary	The most recent data dictionary is posted on the CPCSSN website:<br>http://cpcssn.ca/research-resources/cpcssn-data-dictionary-and-erd/
**Data Processing / Data Provenance**	
Data extraction specifications	The general procedure for EMR data extraction in both SAPCReN and NAPCReN is as follows:<br>▪ Access the EMR frontend or backend, either through the vendor or by a CPCSSN data manager<br>o **Wolf & PS Suite:** backend extraction completed by Telus Health<br>o **Accuro & Healthquest:** backend extraction conducted by a CPCSSN data manager using a log-in account for both clinic and EMR database<br>o **Med Access:** frontend extraction by a CPCSSN data manager using a log-in account created by the clinic<br>▪ Select data tables and columns of interest, then:<br>o Null any columns with identifiable information (e.g. names, addresses, health care numbers)<br>o Select patients with an assigned CPCSSN provider or for those who have not been assigned any provider, use the four-cut method [31] to assign a provider<br>o An EMR mapping file is generated which consists of four data items: EMR patient ID (number assigned by each clinic EMR system), Network ID (number that uniquely identifies each CPCSSN regional network), Site ID (number that uniquely identifies each clinic within a network) and CPCSSN patient ID (unique patient number randomly assigned by CPCSSN). This file is used to ensure CPCSSN patient IDs are assigned uniquely to each EMR patient ID within each clinic and is held separately from the patient health data. The file can also be used to assist with re-identification for practice quality improvement or linkage with other data sources<br>▪ Transfer data to CPCSSN computers via a secure transfer method (e.g. SFTP)

	See Figure 2 for CPCSSN data pipeline.
Mappings from original values to standardized values	The CPCSSN database includes both original data extracted from the EMR, and cleaned or coded information resulting from CPCSSN's algorithms (see CPCSSN Data Dictionary [28]). All original data are extracted from the EMR unchanged, except for a small number of EMR-specific code conversions and some parsing.<br><br><u>General cleaning steps</u><br>▪ Null empty strings and remove leading or trailing whitespace from all records<br>▪ Normalize postal codes to format A1A 1A1<br>o NAPCReN: truncate to forward sortation area code (A1A)<br>▪ Convert all dates to format yyyy-mm-dd<br>▪ Verify code formats for ATC, ICD-9, and DIN classification systems<br>▪ Delete invalid labs where<br>o lab name is a purely numeric value, or matches a text patterns that indicates it is not a useful lab name (e.g. 'unit', 'patient name', 'date')<br>o lab value non-numeric; exceptions are lab values starting with a comparison operator and Hepatitis C<br>▪ Convert medication dispensed forms and duration units to CPCSSN standard forms<br>▪ Delete empty or duplicate records<br>▪ Remove orphaned records (e.g. records with no matching Patient_ID in the Patient table)<br>▪ Remove patients who only have demographic information and no other data<br>▪ Parse billing records that contain multiple conditions into individual records<br><br><u>Allowable ranges of values for NAPCReN and SAPCReN:</u><br>▪ SBP: 50 to 300 mmHg<br>▪ DBP: 20 to 200 mmHg<br>▪ Weight: 1 kg to 500 kg<br>▪ Height: 30 cm to 230 cm<br>▪ BMI (adult): 5 to 200 kg/m^2<br>▪ Waist circumference: 10 to 300 cm<br>▪ Waist-to-hip ratio: 0.1 to 1<br>▪ PEFR: 0-200 L/min<br>▪ Ranges for laboratory values are specific to each of the 44 lab results processed by CPCSSN<br>▪ Age at onset: ≤120<br>▪ Birth year: extraction year (e.g. 2018) minus 120 years<br>▪ Record creation date: earliest EMR date (1990-01-01) to extraction end date (2018-12-31)<br>▪ Condition onset or procedure performed date: 1898-12-31 (extraction end date minus 120 years) to 2018-12-31<br><br><u>Coding to reference system</u><br>▪ **Medication**: existing code, DIN, or free text mapped to codes in the ATC Classification System<br>▪ **Laboratory values:** existing code or free text mapped to LOINC; free-text mapping is completed for 44 labs<br>▪ **Diagnostic codes:** ICD-9 or other codes (e.g. ICD-10, ICPC) found in Health Condition/Profile, Encounter Diagnosis, and Billing tables mapped to ICD-9 codes/classification heading<br>▪ **Diagnostic text:** Pattern matches are used to map free text to ICD-9 codes; this mapping can be completed for 48 conditions in the Health Condition/Profile, Encounter Diagnosis, Billing tables<br>▪ **Referrals**: original text in Referral field mapped to SNOMED concept code; not extracted from referral letters or any PDF documents<br>▪ **Risk factors:** information in the Risk Factor table categorized by text pattern matching to one of the following headings: Smoking, Alcohol, Exercise, Diet, Obesity, or Psychosocial Stress.

	Smoking and Alcohol information further parsed (when available) into Status, Dose, Frequency, Duration, End date<br>▪ **Family history:** relationship type is identified by pattern matching as one of: Mother, Father, Daughter, Son, (Half) Sister, (Half) Brother, (Great) Grandmother, (Great) Grandfather, (Great) Aunt, (Great) Uncle, Niece, Nephew, Cousin, or None. Relationship side (Maternal or Paternal) is also identified by text pattern matching
Data management organization's data transformation routines, including constructed variables	CPCSSN Data Dictionary [28] highlights CPCSSN-constructed variables in blue, including:<br>▪ **Calculated age:** current year minus year of birth *(only as part of the national data provided to approved data users)*<br>▪ **BMI**: uses original BMI if available; otherwise, CPCSSN algorithms generate BMI from original height and weight data where none previously exists<br>▪ **Deprivation category**: maps dissemination areas (geographical units linked to postal code) to material and social quintiles [29] *(for future use in late 2019)*<br>▪ **Disease case and disease case indicator tables**: CPCSSN has developed and validated case definitions based on combinations of diagnoses, billing codes, medications and lab results; patients are indexed according to these definitions. Definitions for conditions currently include: diabetes, hypertension, depression, osteoarthritis, chronic obstructive pulmonary disease, dementia, parkinsonism, epilepsy, plus chronic kidney disease, herpes zoster, and pediatric asthma [5, 6, 14]<br><br>CPCSSN redacts several types of text that could potentially identify patients, providers, or clinics.<br>▪ Non-medical words that may identify specific CPCSSN patients and providers are compared against exclusion lists, containing common words and/or allergy, diagnosis, and/or medication-specific terms to determine which are to be redacted. The exclusion list(s) to apply depend on the type of data being deidentified. Only those names not in the exclusion list(s) will be redacted in free text.<br>▪ Other identifiers, such as social insurance numbers, province health numbers, driver's license numbers, credit card numbers, Worker's Compensation Board numbers, email addresses, and phone numbers (international, North American, and local) are suppressed
Data processing validation routines	During **extractions**, the CPCSSN data are verified by:<br>▪ Comparing the number of files and data volume between extraction cycles occurring every six months<br>▪ Patient and provider counts<br>▪ Table row counts<br>▪ Counts for CPCSSN-specific labs and exams<br><br>During the **data transformation**, a time-stamped log-file provides an itemized description of all processing steps completed, including:<br>▪ Initial and final row counts for all tables<br>▪ Type(s) of transformation(s) and number of data elements transformed<br>▪ Warnings and error messages (e.g. tables missing, processes that cannot be completed)<br><br>During the **coding and cleaning processes**, the following validation tests are performed:<br>▪ Time-stamped log-file which includes an itemized list of completed processes and warnings where data should be coded or is outside allowable ranges<br>▪ Coder process verification tests used to compare the effect of software changes on specific coding or cleaning processes (e.g. ATC coding, exam data cleaning, deidentification) pre- and post-change<br>▪ End-to-end test for case definition integration that compares changes in the validity metrics (i.e. sensitivity, specificity) for CPCSSN case definitions after any changes to any part of the coding and cleaning software

Audit trail	Separate time- and date-stamped log files are retained for each of the extraction, transformation, and cleaning/coding stages.<br>▪ **Extraction**: one file per clinic; contains lists of tables and columns extracted and any warnings or errors that occurred (e.g. connection lost, table missing). An introspection table is created detailing the properties of the source database (e.g. table/column names, data types).<br>▪ **Transformation**: one file per clinic; contains software version information, file locations, initial and final row counts for all tables, type(s) of transformation(s) and number of data elements transformed, warnings and error messages (e.g. tables missing, processes that cannot be completed)<br>▪ **Coding/Cleaning**: one per PBRN; includes an itemized list of processes completed, warnings where data cannot be coded or is outside of allowed ranges
